# Ozone is associated with cardiopulmonary and stroke emergency hospital visits in Reykjavík, Iceland 2003–2009

**DOI:** 10.1186/1476-069X-12-28

**Published:** 2013-04-08

**Authors:** Hanne Krage Carlsen, Bertil Forsberg, Kadri Meister, Thorarinn Gíslason, Anna Oudin

**Affiliations:** 1 Centre of Public Health, University of Iceland, Stapi v/Hringbraut, Reykjavik, 101, Iceland; 2 Occupational and Environmental Medicine, Department of Public Health and Clinical Medicine, Umeå University, Umeå, 901 87, Sweden; 3 Occupational and Environment Medicine, Department of Public Health and Clinical Medicine, University of Gothenburg, Gothenburg, 40530, Sweden; 4 Faculty of Medicine, University of Iceland, Vatnsmyrarvegur 16, Reykjavik, 101, Iceland; 5 Department of Allergy and Sleep (E6), Landspitali University Hospital, Reykjavik, 108, Iceland

**Keywords:** Air pollution, Stroke, Cardiopulmonary, Cardiac, Cardiovascular, Hospital admissions, Emergency room visits

## Abstract

**Background:**

Air pollution exposure is associated with hospital admissions and emergency room visits for cardiopulmonary disease and stroke. Iceland’s capital area, Reykjavik, has generally low air pollution levels, but traffic and natural sources contribute to pollution levels. The objective of this study was to investigate temporal associations between emergency hospital visits and air pollutants ozone (O_3_), nitrogen dioxide (NO_2_), and particulate matter (PM_10_) in the Icelandic capital area.

**Methods:**

We constructed a time series of the daily number of adults who visited the emergency room, or were acutely admitted for stroke or cardiorespiratory causes to Landspitali University Hospital 1 January 2003 – 31 December 2009 from the hospital in-patient register. We used generalized additive models assuming Poisson distribution, to analyze the daily emergency hospital visits as a function of the pollutant levels, and adjusted for meteorological variables, day of week, and time trend with splines.

**Results:**

Daily emergency hospital visits increased 3.9% (95% confidence interval (CI) 1.7-6.1%) per interquartile (IQR) change in average O_3_ the same and two previous days. For females, the increase was 7.8% (95% CI 3.6-12.1) for elderly (70+), the increase was 3.9% (95% CI 0.6-7.3%) per IQR increase of NO_2_. There were no associations with PM_10_.

**Conclusions:**

We found an increase in daily emergency hospital visits associated with O_3_, indicating that low-level exposure may trigger cardiopulmonary events or stroke.

## Background

Iceland's capital Reykjavík is located in the southwest corner of Iceland (Additional file 1) and is the world's most northerly capital. Though air quality is overall good, Iceland has one of the highest per capita car ownership rates and the capital area occasionally experiences high levels of traffic pollutants.

Ozone (O_3_) is a secondary pollutant, with precursors emitted especially from combustion engines. The concentration of O_3_ in Iceland peaks in early spring and Iceland lacks the summer smog commonly associated with O_3_ in the world's larger and more southerly metropolitan areas. The correlations of O_3_ and other pollutants are unusually low in Iceland, for example PM_10_ (particulate matter with an aerodynamic diameter less than 10 μm) and O_3_ are nearly uncorrelated [1]. Ozone has in a few studies been associated with daily mortality, hospital admission for respiratory disease, myocardial infarctions and stroke [2-6]. No study of this kind as previously been done in Iceland.

Particulate matter (PM) concentrations sometimes spike in winter due to primary combustion particles, use of studded tires, and salt and sand spread on roads and sidewalks. Also, sandstorms from areas with little vegetation in southern and central Iceland contribute to PM levels in the capital area [7]. A number of studies have found associations between health and PM levels [8,9], and PM_10_ from sandstorms is associated with cardiorespiratory mortality and hospital admissions [10,11]. In Iceland, associations between PM_10_ and anti-asthma drug sales were found [1].

Nitrogen dioxide (NO_2_) is present in vehicle exhaust, and is used as an indicator of traffic pollutants. NO_2_ is known to be harmful to respiratory health and is associated with exacerbated allergic response in asthmatics [12] and increased rates of asthma emergency room (ER) visits [13]. In Iceland, associations have been found between NO_2_ and angina pectoris symptom-relieving drug sales [14].

The aim of this study was to investigate the associations between urban air pollutants and daily emergency hospital visits for cardiopulmonary disease and stroke, in Iceland's capital area.

## Methods

### Health and environmental data

Pollution data and meteorological data from an urban roadside measuring station for the years 2003 through 2009 were provided by the Environmental Branch of the Municipality of Reykjavík (2003–2008) and the Icelandic Environmental Protection Agency (2009). Unfortunately, the finer fraction of PM_10_, PM_2.5_ (PM < 2.5 μm) measurements were too incomplete to include in the analysis. We investigated the following pollutants: PM_10_, O_3_, and NO_2_. The measuring station was located in Reykjavik (Additional file 1). When the data (30 or 60 minute averages) were at least 75% complete we calculated the 24 hour mean of PM_10_ and NO_2_, for O_3_ we calculated the daily maximum 8-hour mean (commonly used for regulatory purposes).

Data on emergency hospital visits, that is, ER visits and acute hospital admissions to the Landspitali University Hospital in Reykjavik were extracted from the Register of Hospital-treated Patients in Iceland (SAGA) for the period January 2003 to December 2009 (Additional file 2). The Landspitali University Hospital is the only acute hospital care center in the capital area. Data were recorded in the same electronic system throughout the research period. The pulmonary and stroke departments have been operating in the same location throughout the period, but the cardiac ER moved to another location of the unit in 2008. As a primary outcome measure, we selected acute hospital visits of patients who had been admitted to relevant, pre-specified wards and clinics within the hospital, or visited the ER, for one of the following classes of disease: pulmonary disease (ICD10 codes starting with J20-J22, J40-J46 and J96), cardiac disease (ICD10 codes starting with I20-I27, I46, I48 and I50) and cerebrovascular events (ICD10 codes starting with I61-I69 and G45-G46) denoted stroke henceforth. The daily number of emergency room visits and acute hospital admissions were pooled and are known jointly as emergency hospital visits in the following. Data were restricted to patients 18 years or older at the time of the events.

### Statistical analysis

We applied Generalized Additive Models (GAMs), assuming that daily emergency hospital visits were Poisson distributed, allowing for overdispersion. We restricted the study population to individuals with a legal address in the Reykjavík area (postcodes 101 through 270), approximately 200.000 persons (63% of the Icelandic population) due to the location of the pollution measuring station. We modeled the outcomes; all emergency hospital visits (emergency room visits and acute hospital admissions), and stratified for sex and age (cuf-off: 70 years) as a time series. The age cut-off was chosen because at that age most persons in Iceland are retired. When modeling associations where the outcome is delayed relative to the exposure, the model can include lags, for example the exposure the day before the outcome (lag 1) or the average of the same day, and up to two days before (lag 0–2). Previous studies have observed effects of O_3_ at different lags; lag 0 and 1 (effects the same day and the day after the observed exposure) for ischemic stroke [5], lag 3 for recurrent cardiac and cerebrovascular ischemic events [6]; lag 0–3 for COPD admissions [15], lag 1–5 for cardiovascular mortality and morbidity [16]. After careful review of the literature, we decided to use lag 0–2 (average of the levels on the same day, one day, and two days before) for all air pollution variables. As a sensitivity analysis we repeated the main analysis with exposure as same day pollution (lag 0) to the mean of the same day and the previous 5 days (lag 0–5).

We modeled the outcomes as a function of air pollutants in single-, two-, and three-pollutant models. Four outcomes were considered in this study, first all emergency hospital visits, then stratified by sex, and finally emergency hospital visits in elderly (>70 years of age), All models were adjusted for temperature and relative humidity using lag 0–2. The linearity of the association between the outcome and pollution variables were tested with splines and found to be not different from linear (p>0.20). Pollen and influenza epidemic variables were tested in the models, but were not statistically significant, nor did they markedly alter the coefficient estimates for the exposure variables, and were thus excluded from the final models. All models were adjusted for day-of-week and public holidays using binary indicator variables. We then used a cubic, penalized smoothing spline with 8 degrees of freedom for time trend and found that the coefficients were unaltered, but the fit was slightly improved. The models were optimized with respect to deviance explained and visual inspection of the spline functions and the autocorrelation function of the residuals. After including the number of emergency hospital visits at lag 1 as a variable, the autocorrelation function of residuals was found to be reasonable (Additional file 3). As a sensitivity analysis, the three-pollutant model for emergency hospital visits was stratified for season, where summer was defined as April 16 to October 15, and winter October 16 to April 15. In a sensitivity test of high exposure values, especially for PM_10_, we analyzed the data excluding the 5th percentile highest values of the pollutants.

The results from the statistical analysis are reported as change in daily emergency hospital visits (in percent, %) for the interquartile (IQR) change in pollutant concentration. We considered *p*-values under 0.05 to be statistically significant. Data were prepared in PASW Statistics version 18 [17], and analyzed using the 'mgcv' package in R statistical software, version 2.14 [18].

The study was approved by the Science Bioethics Committee (VSNb2010120017/03.7), the Data Protection Agency (2010121176AT/--) and the Landspitali University Hospital Medical Executive board (December 22nd 2010).

## Results

**Figure 1:**
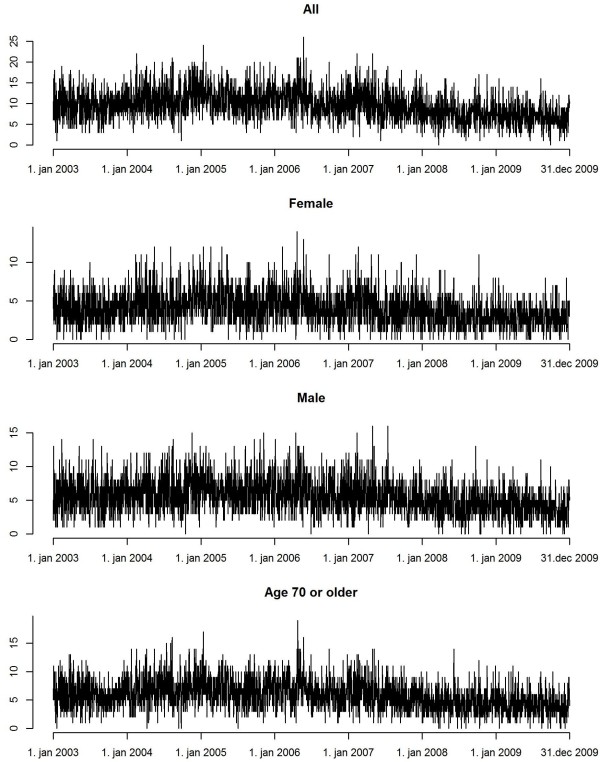
Daily emergency hospital visits for cardiopulmonary, respiratory and stroke diagnoses to the Landspítali University Hospital in Reykjavík

During the 7-year period, there were 24439 emergency hospital visits, of which the majority (76.9%) was for cardiac causes, whereas 16.7% were for stroke and 6.4% for pulmonary causes (Table 1, Figure 1). The mean daily number of emergency hospital visits was lower towards the end of the study period. Emergency hospital visits for cardiac causes dropped from on average 8 per day during 2003–2007 to circa 6 in 2008–2009, coinciding with the move of the cardiac ER to a separate location from the other ER departments.

**Table 1:** Daily emergency hospital visits, pollutant levels and meteorological variables in Reykjavík 2003-2009

	**n (%)**	**Mean**	**Std. dev**	**Min-imum**	**Max-imum**	**Percentiles**		
						**25th**	**50th**	**75th**
**Emergency hospital visits**								
All	24439 (100%)	9.6	3.8	0	26	7	9	12
Cardiac^a^	18782 (76.9%)	7.3	3.3	0	20	5	7	9
Stroke^b^	4082 (16.7%)	1.6	1.3	0	8	1	1	2
Pulmonary^c^	1575 (6.4%)	0.6	0.8	0	5	0	0	1
Female	10269 (42.0%)	4.0	2.2	0	14	2	4	5
Male	14170 (58.0%)	5.5	2.6	0	16	4	5	7
Age 70 years or older	14862 (60.8%)	5.8	2.7	0	19	4	6	7
**Pollutants & meteorological variables**								
O_3_ (μg/m^3^)^d^	2355 (92.1%)	47.3	16.8	1.4	119.4	36.3	46.1	57.2
NO_2_ (μg/m^3^)	2372 (92.8%)	22.1	13.6	1.5	111.6	12.0	19.2	28.6
PM_10_ (μg/m^3^)	2396 (93.7%)	21.2	19.8	1.3	261.1	11.0	15.6	23.6
Temperature (°C)	2533 (99.1%)	6.0	5.2	−11.3	21.2	2.3	6.2	10.4
Relative humidity (%)	2533 (99.1%)	78.0	10.8	39.3	100.0	70.5	79.6	86.3

Daily counts for emergency hospital visits were available for all days. n denotes the total number of emergency hospital visits. For pollutants and meteorological variables, n denotes the number of days with data available. The total number of days in the period is 2557.

Std. dev; Standard deviation.

^a^ Cardiac disease (ICD10 codes starting with I20-I27, I46, I48 and I50).

^b^ Stroke (Cerebrovascular disease) (ICD10 codes starting with I61-I69 and G45-G46).

^c^ Pulmonary diseases (ICD10 codes starting with J20-J22, J40-J46 and J96).

^d^ Maximum daily 8-hour mean, daily 24-hour mean for other exposure variables.

**Figure 2:**
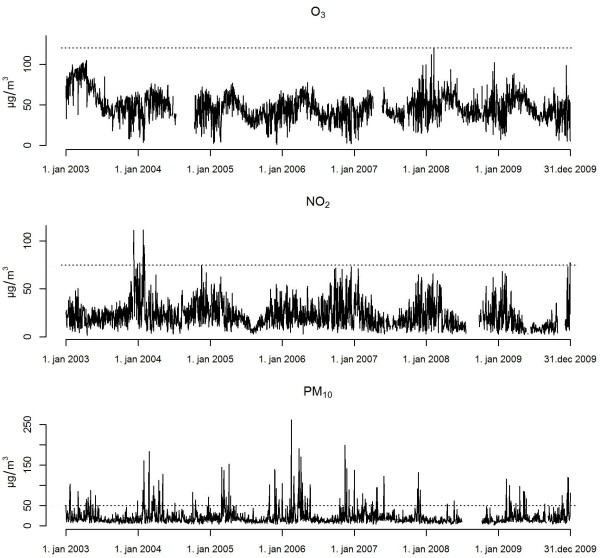
**Daily pollutant levels in Reykjavík 2003–2009, 24-hour means of NO**_**2 **_**and PM**_**10 **_**, and the 8-hour maximum of O**_**3**_**.** The dotted lines denote the relevant health limits.

The pollution data were at least 92.1% complete (Table 1). NO_2_ and O_3_ exhibit distinct seasonality, PM_10_ have relatively low background levels, but high, intermittent peaks, which are often associated with meteorological factors, sandstorm activity and special events, such as New Year's Eve where abundant amounts of fireworks are fired every year. The mean annual temperature during the study period was 6.0°C (Table 1, Figure 2).

The correlations between most pollutants and meteorological variables were moderate, except the correlation between O_3_ and NO_2_, r=−0.47 (p<0.001) and temperature and NO_2_, r = −0.43 (p<0.001: Table 2).

**Table 2:** Correlation coefficient matrix of the exposure variables (Pearson)

	**O**_**3**_^**a**^	**PM**_**10**_	**NO**_**2**_	**Temperature**	**Relative humidity**
**O**_**3**_^**a**^	1	0.03	−0.47^#^	−0.03	−0.12^#^
**PM**_**10**_		1	0.16^#^	−0.26^#^	−0.32^#^
**NO**_**2**_			1	−0.43^#^	0.07^#^
**Temperature**				1	0.11^#^
**Relative humidity**					1

^a^ Maximum daily 8-hour mean, daily 24-hour mean for other exposure variables.

P <0.001:#.

In the single pollutant analysis, an IQR increase in O_3_ levels was associated with an increase in all emergency hospital visits with 3.9% (95% CI 1.7 – 6.1%, p<0.01; Table 3). Association with NO_2_ were not statistically significant, Associations with PM_10_ were close to null. In the three-pollutant analysis; (NO_2_, O_3_ and PM_10_ in the same model) we found that an IQR change in O_3_ was associated with an increase in emergency hospital visits of 5.3% (2.5 - 8.1%, p<0.01). No associations with PM_10_ were found (Table 3). The O_3_ and PM_10_ effect estimates from two-pollutant models were similar to the effect estimates from the single-pollutant models, but the NO_2_ estimates were altered (Additional file 4: Table A).

**Table 3:** Associations between daily emergency hospital visits and pollution levels (lag 0–2) in single-and three pollutant models

	**O**_**3**_		**NO**_**2**_		**PM**_**10**_	
	**% (95% CI)**	***p***	**% (95% CI)**	***p***	**% (95% CI)**	***p***
**All**						
Single-pollutant	3.9 (1.7-6.1)	<0.01	−1.4 (−3.3-0.6)	0.17	0.1 (−0.9-1.2)	0.86
Three-pollutant	5.3 (2.5-8.1)	<0.01	1.5 (−1.0-4.1)	0.24	−0.4 (−1.7-0.7)	0.45
**Females**						
Single-pollutant	5.7 (2.4-9.1)	<0.01	−1.6 (−4.5-1.4)	0.29	0.1 (−1.5-1.8)	0.86
Three-pollutant	7.8 (3.6-12.1)	<0.01	2.7 (−1.1-6.8)	0.17	−0.6 (−2.4-1.2)	0.51
**Males**						
Single-pollutant	2.9 (0.1-5.8)	0.04	−1.1 (−3.7-1.5)	0.39	0.1 (−1.3-1.5)	0.88
Three-pollutant	3.5 (0.1-6.9)	0.04	0.6 (−2.6-3.9)	0.73	−0.1 (−1.7-1.4)	0.86
**Elderly**						
Single-pollutant	3.3 (0.5-6.1)	0.02	−0.1 (−2.6-2.5)	0.96	0.0 (−1.4-1.4)	0.98
Three-pollutant	6.5 (3.0- 10.1)	<0.01	3.9 (0.6-7.3)	0.02	−0.6 (−2.2-0.9)	0.43

Results are given as percent (%) change in visits per interquartile range (μg/m^3^) increase. Models are adjusted for lag 1 of relevant visits, day-of-week and odd holidays, lag 02 temperature and relative humidity, and time trend with a cubic-penalized spline. Interquartile range of lag 02 variables: O_3_: 17.21 μg/m^3^, NO_2_: 12.93 μg/m^3^, PM_10_: 11.05 μg/m^3^.

Stratifying the emergency hospital visits by sex, the effect estimates for O_3_ were higher in women than in men; female emergency hospital visits increased 7.8% (95% CI 3.6 – 12.1%, p<0.01) with an IQR increase in O_3_, whereas the increase was 3.5% (95% CI 0.1 - 6.9%, p=0.04) for men. Associations with PM_10_ in males and females were close to null. In elderly (those aged 70 or older), we found that the number of emergency hospital visits increased with 6.5% (95% CI 3.0 – 10.1%, p<0.01) per IQR increase in O_3_, and 3.9% (95% CI 0.6 – 7.3%, p = 0.02) per IQR increase in NO_2_ (Table 3).

For all models, the lag 1 variable; emergency hospital visits of the day before, was positively associated with the outcome. This was most pronounced in those 70 years or older. The effect estimates were only slightly affected by changes in the length of the lag (Additional file 4: Table B). Excluding high values, or stratification by season did not alter the point estimates associated with O_3_ substantially (data not shown).

## Discussion

In the study, which was undertaken in a low-pollutant urban area, cardiopulmonary and stroke emergency hospital visits were associated with O_3_ levels of the same day and the previous two days. We found that the effect estimate was higher in women than men. In those aged 70 years or older, we observed higher associations with O_3_ and there seemed to be an effect also from NO_2_. We observed no effect of PM_10_ in this setting. These results suggest that worsening of respiratory disease, cardiac events and stroke could be triggered by short term exposure to increased O_3_ levels, and that women and the elderly could be more susceptible.

A review of cardiovascular injury and O_3_, [19] found a likely association in both epidemiological studies and in vitro studies of inflammatory markers. A case-crossover study of dispensing of quick-acting drugs for angina pectoris symptoms (nitroglycerines) found associations with same-day and lag 1 of O_3_ and NO_2_ levels in Iceland’s capital area [14]. The estimated effects observed in the present study were similar to those from a study of O_3_ levels and incident ischemic events, stroke and myocardial infarction. Here, the association was modified by underlying diseases [5,6]. O_3_ effects are mainly found in elderly and inflammation and vasoconstriction of the arteries are suspected underlying mechanisms for cardiovascular outcomes, while oxidative irritation has been suggested for respiratory effects [16]. Unfortunately, we had no data on comorbidities, but the O_3_ and NO_2_ effect estimate were higher in the elderly (≥ 70 years of age) who are more likely to have underlying diseases than younger persons. Previous studies have found sex-differentiated effect of O_3_; associations with ischemic stroke in men, but not in women [5], and higher associations with mortality in women than men [20]. Moreover, O_3_ exposure exacerbated pneumonia in female rats, but not in male rats [21]. Unfortunately the proportion of stroke events and pulmonary admissions in the present study were too few to analyze separately stratified by sex. Some findings suggest that O_3_ effects appear mostly in summer [4], probably because O_3_ peaks when temperatures are high in urban areas in warmer climates. In Finland, higher summer effects were found [16]. However, a large European study found strong associations between ozone and daily mortality in winter, after adjusting for the negative correlation between O_3_ and primary combustion products (e.g. CO) [3]. In the present study, effect estimates for O_3_ were similar when stratified by season, somewhat expected, as the seasonal temperature variation is limited (data not shown).

In our study, NO_2_ was only associated with our outcome in those 70 years or older. As the majority of the emergency hospital visits were due to cardiac causes, this is consistent with the association in the elderly observed in previous studies where associations have been observed between NO_2_ and admissions for cardiorespiratory causes, especially in the elderly [13], and NO_2_ levels and angina pectoris medication sales [14]. We observed no association between PM_10_ and emergency hospital visits, although PM_10_ was the only variable which significantly exceeded the official health limits values in our data. The 24-hour health limits for air pollution in Iceland are 50 μg/m^3^ for PM_10_, 75 μg/m^3^ for NO_2_, and 120 μg/m^3^ for 8-hour O_3_. Several studies have shown health effects from coarse particles [22] and studies have found associations with mortality in Stockholm [8] and also associations with respiratory admissions [23]. Positive associations between ischemic stroke and PM_10_ were found by Oudin and colleagues [24]. However, strokes were only a small fraction of the emergency hospital visits in this study. It was a limitation of the present study that no data on fine particles, PM_2.5_, were available. Associations between fine particles and admissions for respiratory- and cardiovascular disease have previously been shown [23,25,26]. Also, Peng and colleagues [23] found that small associations between PM_2.5–10_ levels and admissions in individuals 65 years or older for cardiovascular- and respiratory causes disappeared after adjusting the results for PM_2,5_. The low risk associated with increased levels of PM_10_ could be related to that PM at high levels may originate at other sources than at lower levels. Particle composition also influences particle toxicity. As the Icelandic capital area is surrounded by sea on three sides the salt content in PM is substantial [27]. In a study of cardiovascular admissions and PM_2.5_, no associations with particles containing salt components were found [26], neither between mortality and aged sea salt particles [28]. In a study of cardiorespiratory admissions in children and elderly there were no observed associations between PM salt content and admissions, and it was suggested that salt could either act as a proxy of cleaner air (sea salt) or road dust (road salt) [29]. A high proportion of sea salt in PM_10_ could be a factor the non-observed association with PM_10_ in this study. Unfortunately, no information is currently available on the daily variation of sea salt in PM in our study area.

We found positive associations between relative humidity and emergency hospital visits and PM_10_ was negatively correlated with relative humidity (r=−0.33), which could modify the PM_10_ effect on emergency hospital visits. However, when excluding relative humidity from the model, PM_10_ effect estimate did not change. In the sensitivity analysis excluding the highest values from the data set, only the PM_10_ effect estimate changed noticeably, from negative to positive (data not shown), indicating that the very high values of PM_10_ are independently associated with our health outcome. PM_10_ in Iceland has been found to contain high levels of soil and organic matter [27] which could influence toxicity. We aim to investigate this in future research.

The correlations between O_3_ and PM_10_ and O_3_ and meteorological variables were close to null in our study, whereas in France, higher correlations were found between O_3_ and PM_10_ (r = −.22), and between O_3_ and temperature, (r = .74) [6]. Other studies have found higher correlations between O_3_ and PM_10_, and O_3_ and temperature [16], and substantial variance in the correlation between O_3_, PM_10_ and NO_2_ in different cities in Italy [20]. The low pollutant correlations in the present study are interesting given that we observed associations with O_3_, but not with PM_10_, somewhat in contrast to findings from other studies. This could indicate that something about the pollution mixture or atmospheric conditions are different in this setting.

We combined cardiac, pulmonary and stroke emergency hospital visits in order to get sufficient statistical power, and chose to use lag 0–2 as the exposure to event-time is quite likely different for the three outcomes. A sensitivity analysis of different lag structures revealed lag 0–2 to be a good fit without losing too much data. The results were not sensitive to changes in lag structures (Additional file 4: Table B). The time trend spline had 8 degrees of freedom, when using higher number of degrees of freedom, the autocorrelation- and partial autocorrelation functions showed signs of overfitting.

The cardiac emergency department moved during the study period, but there was no change in the number of visits per day outside of an already downwards trend. A sensitivity analysis of the period before and after also revealed no significant changes. Data from one measuring station were used to estimate exposure for the entire study population in this study, and while inaccurate exposure assignment across the study area can cause a non-differential error, it is unlikely to skew the results, but decreases the statistical power [30]. The ecological time series design is independent of personal risk factors which do not change over time, underlying disease, and recurrent or incident events. While the study design does not allow us to further explore effect modification due to personal risk factors, other studies have shown increased risks in people with underlying disease [6,12].

## Conclusions

In this study of daily acute hospital admissions and emergency room visits for cardiopulmonary and stroke events, we observed a positive association with pollutant concentrations of O_3_. The effect estimates were higher in women and in the elderly. In elderly we also found a positive association with NO_2_. There were no associations with PM_10_. The findings indicate that in spite of moderate O_3_ levels and the lack of a summer ozone peak, O_3_ may trigger emergency hospital visits.

## Consent

According to the legislation of Iceland, no informed consent from participants was needed because data were analyzed anonymously.

The study was approved by the Bioethics Committee (VSNb2010120017/03.7), the Data Protection Agency (2010121176AT/--)and the Landspítali University Hospital Medical Exedutive Board (22 December 2010).

## Abbreviations

CI: Confidence Interval; CO: Carbon monoxide; ER: Emergency Room; GAM: Generalized Additive Model; IQR: Interquartile Range; NO2: Nitrogen dioxide; O3: Ozone; PM: Particulate Matter.

## Competing interests

The authors declare no competing financial interests or other conflicts of interest.

## Authors’ contributions

HKC, TG and AO collected the data, HKC, AO, KM and BF analysed the data. The manuscript was primarily written by HKC and AO, TG, KM and BF provided essential input and feedback. All authors read and approved the final manuscript.

## Supplementary Material

Additional file 1: Figure A Map of Iceland (insert) and the capital area with urban areas, major roads and pollution sources indicated. Click here for file

Additional file 2: Figure B Flow-chart of the study population selection process. Click here for file

Additional file 3: Figure C Diagnostic plots for the model of all emergency hospital visits and lag 0–2 of pollutants. Click here for file

Additional file 4: Table A Two-pollutant models. Associations between daily emergency hospital visits and pollution levels (lag 0–2). Results are given as percent (%) change in visits per interquartile range (μg/m^3^) increase. **Table B.** Sensitivity analysis. Outcomes from three-pollutant regression models with different lag structures and identical covariates. Results are given as percent (%) change in visits per 10 μg/m^3^ pollutant increase. Click here for file

## References

[B1] Carlsen HK Zoëga H Valdimarsdóttir U Gíslason T Hrafnkelsson B Hydrogen sulfide and particle matter levels are associated with increased dispensing of anti-asthma drugs in Iceland’s capital Environ Res 2012 113 33 39 22264878 10.1016/j.envres.2011.10.010

[B2] Anderson HR Atkinson R Peacock J Marston L Konstantinou K Meta-analysis of time-series studies and panel studies of particulate matter (PM) and ozone (O3). report of a WHO task group 2004 WHO www.euro.who.int/__data/assets/pdf_file/0004/74731/e82792.pdf

[B3] Gryparis A Forsberg B Katsouyanni K Analitis A Touloumi G Schwartz J Samoli E Medina S Anderson HR Niciu EM Wichmann H-E Kriz B Kosnik M Skorkovsky J Vond JM Dörtbudak Z Acute effects of ozone on mortality from the "Air pollution and health: a european approach" project Am J Respir Crit Care Med 2004 170 1080 1087 10.1164/rccm.200403-333OC 15282198

[B4] Bell ML Dominici F Samet JM A meta-analysis of time-series studies of ozone and mortality with comparison to the national morbidity, mortality, and air pollution study Epidemiology 2005 16 436 445 10.1097/01.ede.0000165817.40152.85 15951661 PMC3581312

[B5] Henrotin JB Besancenot JP Bejot Y Giroud M Short-term effects of ozone air pollution on ischaemic stroke occurrence: a case-crossover analysis from a 10-year population-based study in Dijon, France J Occup Environ Med 2007 64 439 445 10.1136/oem.2006.029306 PMC2078476 17409181

[B6] Henrotin J-B Zeller M Lorgis L Cottin Y Giroud M Béjot Y Evidence of the role of short-term exposure to ozone on ischaemic cerebral and cardiac events: the Dijon vascular project (DIVA) Heart 2010 96 1990 1996 10.1136/hrt.2010.200337 20702540

[B7] Thorsteinsson T Gísladóttir G Bullard J McTainsh G Dust storm contributions to airborne particulate matter in Reykjavík, Iceland Atmos Environ 2011 45 5924 5933 10.1016/j.atmosenv.2011.05.023

[B8] Meister K Johansson C Forsberg B Estimated short-term effects of coarse particles on daily mortality in Stockholm, Sweden Environ Health Perspect 2012 120 431 436 22182596 10.1289/ehp.1103995 PMC3295353

[B9] Oudin A Forsberg B Jakobsson K Exposure to particulate Air pollution triggers recurrent, but Not first-ever, ischemic stroke Epidemiology 2012 23 505 506 10.1097/EDE.0b013e31824ea667 22475837

[B10] Perez L Tobias A Querol X Künzli N Pey J Alastuey A Viana M Valero N González-Cabré M Sunyer J Coarse particles from Saharan dust and daily mortality Epidemiology 2008 19 800 807 10.1097/EDE.0b013e31818131cf 18938653

[B11] Tam WWS Wong TW Wong AHS Hui DSC Effect of dust storm events on daily emergency admissions for respiratory diseases Respirology 2012 17 143 148 10.1111/j.1440-1843.2011.02056.x 22092966

[B12] Barck C Sandström T Lundahl J Halldén G Svartengren M Strand V Rak S Bylin G Ambient level of NO_2_ augments the inflammatory response to inhaled allergen in asthmatics Respir Med 2002 96 907 917 10.1053/rmed.2002.1374 12418589

[B13] Latza U Gerdes S Baur X Effects of nitrogen dioxide on human health: systematic review of experimental and epidemiological studies conducted between 2002 and 2006 Int J Hyg Environ Health 2009 212 271 287 10.1016/j.ijheh.2008.06.003 18771952

[B14] Finnbjornsdottir RG Zoega H Olafsson O Rafnsson V Air pollution in Reykjavík and dispensation of drugs for angina pectoris [abstract] Eur Respir J 2012 40 supplement) P3390

[B15] Fung KY Khan S Krewski D Chen D Association between air pollution and multiple respiratory hospitalizations among the elderly in Vancouver, Canada Inhal Toxicol 2006 18 1005 1011 10.1080/08958370600904538 16966300

[B16] Halonen JI Lanki T Tiittanen P Niemi JV Loh M Pekkanen J Ozone and cause-specific cardiorespiratory morbidity and mortality J Epidemiol Commun Health 2010 64 814 820 10.1136/jech.2009.087106 19854743

[B17] SPSS Inc PASW Statistics for Windows, Version 18.0 2009 Chicago, USA: SPSS Inc

[B18] Team RDC R: a language and environment for statistical computing 2012 Vienna, Austria http://www.R-project.org/

[B19] Srebot V Gianicolo EA Rainaldi G Trivella MG Sicari R Ozone and cardiovascular injury Cardiovasc Ultrasound 2009 7 30 10.1186/1476-7120-7-30 19552797 PMC2706799

[B20] Stafoggia M Forastiere F Faaustini A Trivella MG Sicari R Susceptibility factors to ozone-related mortality: a population-based case-crossover analysis Am J Respir Crit Care Med 2010 182 376 384 10.1164/rccm.200908-1269OC 20339147

[B21] Mikerov AN Cooper TK Wang G Hu S Umstead TM Phelps DS Floros J Histopathologic evaluation of lung and extrapulmonary tissues show sex differences in *Klebsiella pneumoniae* - infected mice under different exposure conditions Int J Physiol Pathophysiol Pharmacol 2011 30 176 190 21941609 PMC3175744

[B22] Brunekreef B Forsberg B Epidemiological evidence of effects of coarse airborne particles on health Eur Respir J 2005 26 309 318 10.1183/09031936.05.00001805 16055881

[B23] Peng RD Chang HH Bell ML McDermott A Zeger SL Samet JM Dominici F Coarse particulate matter air pollution and hospital admissions for cardiovascular and respiratory diseases among Medicare patients J Am Med Assoc 2008 299 2172 2179 10.1001/jama.299.18.2172 PMC3169813 18477784

[B24] Oudin A Strömberg U Jakobsson K Stroh E Björk J Estimation of short-term effects of air pollution on stroke hospital admissions in southern Sweden Neuroepidemiology 2010 34 131 142 10.1159/000274807 20068360

[B25] Peng RD Bell ML Geyh AS McDermott A Zeger SL Samet JM Dominici F Emergency admissions for cardiovascular and respiratory diseases and the chemical composition of fine particle Air pollution Environ Health Perspect 2009 117 957 963 10.1289/ehp.0800185 19590690 PMC2702413

[B26] Pope CA Muhlestein JB May HT Renlund DG Anderson JL Horne BD Ischemic heart disease events triggered by short-term exposure to fine particulate air pollution Circulation 2006 114 2443 2448 10.1161/CIRCULATIONAHA.106.636977 17101851

[B27] Skúladóttir B Larssen S Bjarnason G Hallsdóttir B Gustafsson L Guðmundsdóttir A Methods for determining the composition of airborne particle pollution in Reykjavík 2003 Icelandic Technological Institute

[B28] Ostro B Tobias A Querol X Alastuey A Amato F Pey J Pérez N Sunyer J The effects of particulate matter sources on daily mortality: a case-crossover study of Barcelona, Spain Environ Health Perspect 2011 119 1781 1777 10.1289/ehp.1103618 21846610 PMC3261985

[B29] Andersen ZJ Wahlin P Raaschou-Nielsen O Ketzel M Scheike T Loft S Size distribution and total number concentration of ultrafine and accumulation mode particles and hospital admissions in children and the elderly in Copenhagen, Denmark Occup Env Med 2010 65 458 466 17989204 10.1136/oem.2007.033290

[B30] Zeger SL Thomas D Dominici F Samet JM Schwartz J Dockery D Cohen A Exposure measurement error in time-series studies of air pollution: concepts and consequences Environ Health Perspect 2000 108 419 426 10.1289/ehp.00108419 10811568 PMC1638034

