# Supplementary figures and images for: Ozone is associated with cardiopulmonary and stroke emergency hospital visits in Reykjavík, Iceland 2003–2009
Source: Environ Health. 2013 Apr 8;12:28. doi: 10.1186/1476-069X-12-28 (PMC3639138; doi:10.1186/1476-069X-12-28)

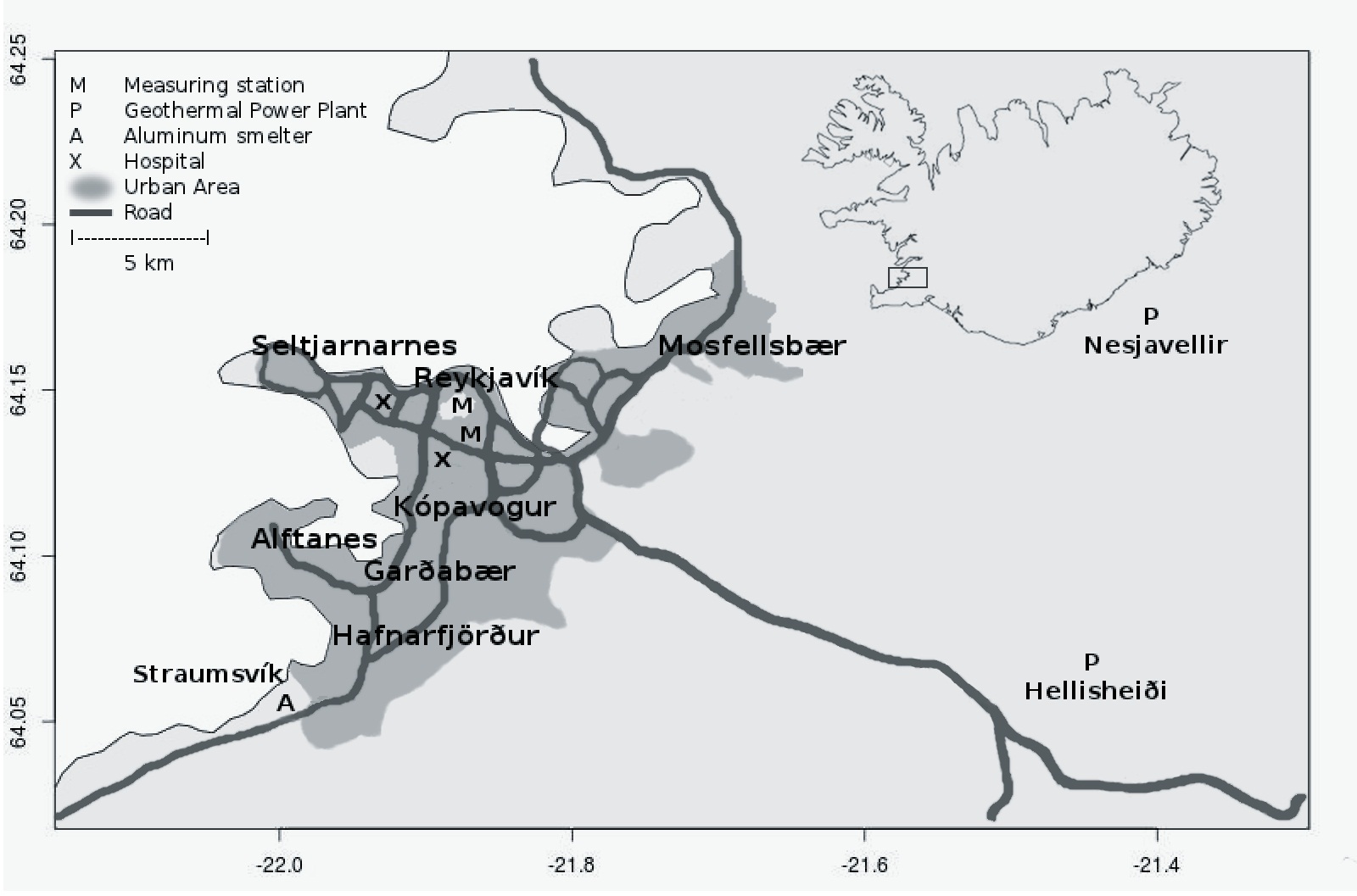

Supplement: Additional file 1: Figure A — Map of Iceland (insert) and the capital area with urban areas, major roads and pollution sources indicated. [file 1476-069X-12-28-S1.jpeg]

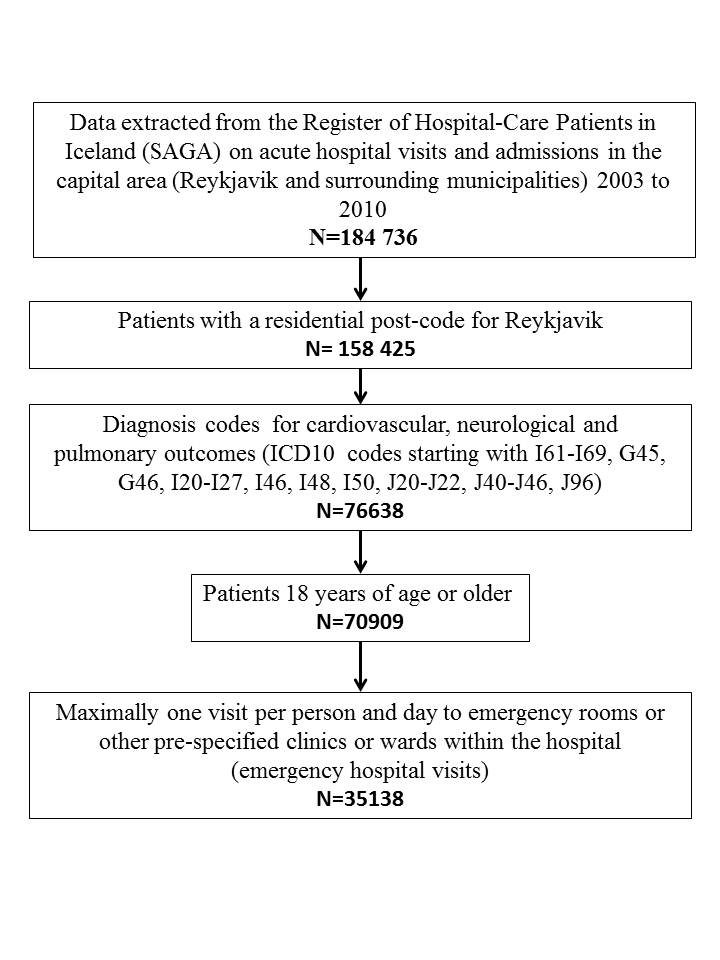

Supplement: Additional file 2: Figure B — Flow-chart of the study population selection process. [file 1476-069X-12-28-S2.jpeg]

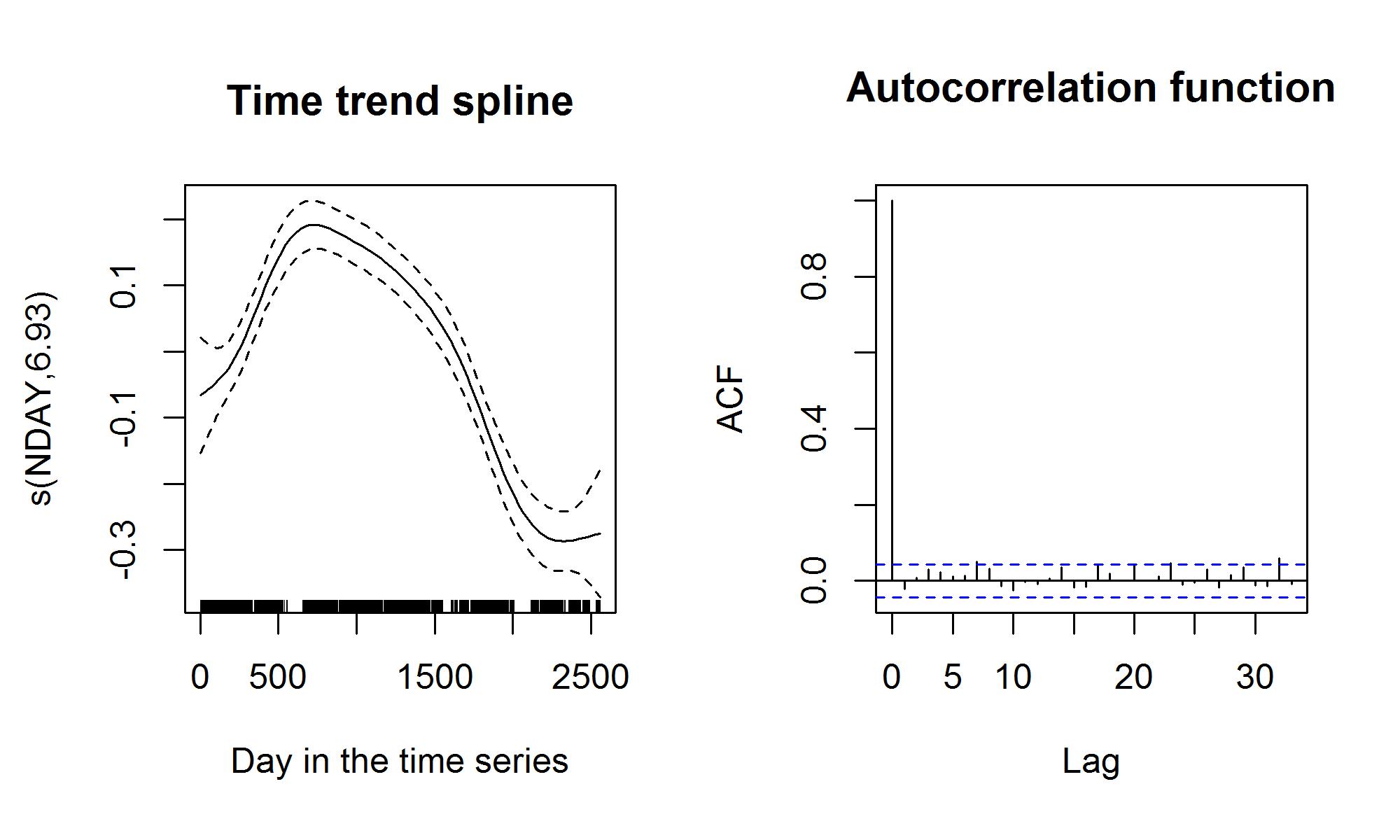

Supplement: Additional file 3: Figure C — Diagnostic plots for the model of all emergency hospital visits and lag 0–2 of pollutants. [file 1476-069X-12-28-S3.jpeg]
