# Supplementary material for: Ozone is associated with cardiopulmonary and stroke emergency hospital visits in Reykjavík, Iceland 2003–2009
Source: Environ Health. 2013 Apr 8;12:28. doi: 10.1186/1476-069X-12-28 (PMC3639138; doi:10.1186/1476-069X-12-28)
Supplement: Additional file 4: Table A — Two-pollutant models. Associations between daily emergency hospital visits and pollution levels (lag 0–2). Results are given as percent (%) change in visits per interquartile range (μg/m3) increase. Table B. Sensitivity analysis. Outcomes from three-pollutant regression models with different lag structures and identical covariates. Results are given as percent (%) change in visits per 10 μg/m3 pollutant increase. [file 1476-069X-12-28-S4.docx]

**Table A Two-pollutant models. Associations between daily emergency hospital visits and pollution levels (lag 0-2). Results are given as percent (%) change per interquartile range (μg/m^3^) increase**

|  | **O_3_** | | **NO_2_** | | **PM_10_** | |
| --- | --- | --- | --- | --- | --- | --- |
|  | % (95% CI) | *p* | % (95% CI) | *p* | % (95% CI) | *p* |
| O_3_ – NO_2_ | 5.3 (2.6, 8.0) | <0.01 | 1.5 (-1.0, 4.1) | 0.23 | - |  |
| O_3_ – PM_10_ | 4.2 (1.9, 6.4) | <0.01 | - | - | -0.2 (-1.4, 0.9) | 0.70 |
| NO_2_ – PM_10_ | - | - | -1.4 (-3.4, 0.6) | 0.16 | 0.0 (-1.1, 1.1) | 0.98 |

The models are adjusted for lag 1 of emergency hospital visits, day-of-week and odd holidays, lag 0-2 temperature and relative humidity, and time trend with a cubic-penalized spline. Interquartile range of lag 0-2 variables: O_3_: 17.21 μg/m^3^, NO_2_: 12.93 μg/m^3^, PM_10_: 11.05 μg/m^3^.

**Table B Sensitivity analysis. Outcomes from three-pollutant regression models with different lag structures and identical covariates. Results are given as percent (%) change per 10 μg/m^3^ pollutant increase**

|  | **O_3_** | | | **NO_2_** | | **PM_10_** | | | Deviance Explained |
| --- | --- | --- | --- | --- | --- | --- | --- | --- | --- |
|  | % (95% CI) | *p* | | % (95% CI) | *p* | % (95% CI) | *p* | |  |
| **Lag 0** | 1.5 (0.2, 2.8) | | 0.02 | 0.9 (-0.6, 2.4) | 0.23 | 0.0 (-0.6, 0.8) | | 0.89 | 30.1% |
| **Lag 0-1** | 2.6 (1.2, 4.1) | | <0.01 | 1.0 (-0.7, 2.8) | 0.25 | -0.3 (-1.2, 0.6) | | 0.51 | 30.3% |
| **Lag 0-2** | 3.0 (1.5, 4.5) | | <0.01 | 1.2 (-0.8, 3.1) | 0.24 | -0.4 (-1.5, 0.7) | | 0.45 | 30.9% |
| **Lag 0-3** | 3.6 (2.0, 5.2) | | <0.01 | 1.4 (-0.7, 3.5) | 0.18 | -0.5 (-1.7, 0.7) | | 0.40 | 30.9% |
| **Lag 0-4** | 4.1 (2.5, 5.8) | | <0.01 | 1.5 (-0.7, 3.7) | 0.19 | -0.8 (-2.1, 0.5) | | 0.26 | 31.1% |
| **Lag 0-5** | 4.3 (2.6, 6.1) | | <0.01 | 1.7 (-0.6, 4.0) | 0.15 | -0.6 (-2.0, 0.8) | | 0.40 | 30.9% |

The models are adjusted for lag 1 emergency hospital visits, day-of-week and odd holidays, lag 0-2 temperature and relative humidity, and time trend with a cubic-penalized spline.
